# How Does Observational Learning Produce Placebo Effects? A Model Integrating Research Findings

**DOI:** 10.3389/fpsyg.2018.02041

**Published:** 2018-10-24

**Authors:** Elżbieta A. Bajcar, Przemysław Bąbel

**Affiliations:** Pain Research Group, Institute of Psychology, Jagiellonian University, Kraków, Poland

**Keywords:** modeling, nocebo hyperalgesia, observational learning, social learning, placebo analgesia

## Abstract

There is a growing body of evidence proving that observational learning, in addition to classical conditioning and verbal suggestions, may induce both placebo analgesia and nocebo hyperalgesia. However, much less is known about the mechanisms and factors influencing placebo effects induced by observational learning. The paper critically reviews the research findings in the field in the context of Bandura’s social learning theory. We apply Bandura’s taxonomy of the sources of social learning (behavioral, symbolic, and verbal modeling) and discuss the results of previous studies. Critical points in the placebo effects induced by observational learning are identified. We discuss aspects of behavior presented by the model (both verbal and non-verbal) involved in the formation of placebo effects induced by observational learning as well as the role of expectancies in this process. As a result, we propose a model that integrates the existing research findings. The model shows the main ways of transmission of pain-related information from the model to the observer. It highlights the role of expectancies and the individual characteristics of the observer in formation of placebo analgesia and nocebo hyperalgesia induced by observational learning. Finally, we propose future research directions based on our model.

## Introduction

There is a large volume of published experimental studies investigating the learning mechanisms of placebo analgesia and nocebo hyperalgesia. These studies have shown that placebo effects may be induced by first-hand pain experiences, i.e., classical conditioning (Colloca and Benedetti, 2006; Colloca et al., 2010; Jensen et al., 2012, 2015; Au Yeung et al., 2014; Bąbel et al., 2017a,b) and verbal suggestions (Benedetti et al., 2007; Colloca et al., 2008; van Laarhoven et al., 2011; Aslaksen and Lyby, 2015). However, Bootzin and Caspi (2002) have also suggested that observational learning may be one of the explanatory mechanisms for placebo effects. Indeed, the behavior of others may be a highly informative source of data about a situation and about the possible reinforcing consequences of a specific action in this situation (Rosenthal and Zimmerman, 1978; Bandura, 1985). This information may contribute to changes in the observer’s patterns of behavior and elicit responses similar to those presented by the model. This process is called observational learning and is the core of the social learning theory formulated by Bandura (1976, 1985). Recently a considerable amount of studies have been published investigating the role of social models in pain modulation (for review see: Goubert et al., 2011), especially in the context of placebo effects (for review see: Faasse and Petrie, 2016; Schenk et al., 2017).

Studies on the role of social factors in pain perception and pain modulation seem to be particularly important because people suffering from pain are usually not only confronted with their own pain experiences but are also strongly influenced by the pain behaviors of others. Benedetti (2013) stresses that the observation of other people by participants in clinical studies can positively or negatively affect obtained results. Patients’ observation of ineffective attempts to treat other patients can have a negative impact on the effects of their own therapy, just as observing effective treatment attempts can have a positive impact on patients’ health. Generally, observation of other ill people in the media or outside a clinical context can positively or negatively influence the observer’s health.

As two recent papers have already excellently summarized the current state of the art in the placebo effects induced by observational learning (Faasse and Petrie, 2016; Schenk et al., 2017), it is now time to go further toward a more theoretical account. Our paper aims to (1) critically review the research findings in the placebo effects induced by observational learning, (2) propose a model integrating the research findings, and (3) propose future research directions based on this model.

## Placebo Effects Induced by Three Types of Observational Learning

According to the social learning theory (Bandura, 1976, 1985), information can be conveyed to observers in three ways: through physical demonstration of specific behavior (behavioral modeling), through indirect pictorial representation (symbolic modeling, e.g., videos), and through verbal description of behavior (verbal modeling). Although authors of studies on the role of observational learning in the placebo effects have not yet used this taxonomy, they have indeed applied these three types of sources of social learning to induce placebo effects. Thus, we will follow this taxonomy when discussing the results of the studies.

Colloca and Benedetti (2009) were the first to investigate the effects of observational learning on placebo analgesia. In their seminal study, they applied the color lights paradigm which was also used in the other experimental studies on placebo effects induced by classical conditioning and verbal suggestions (Colloca and Benedetti, 2006; Colloca et al., 2008, 2010; Lui et al., 2010; Bartels et al., 2014; Bąbel et al., 2017a,b). The female participants watched an unfamiliar male model who was rating as non-painful or painful electrical pain stimuli preceded by green lights or red lights, respectively (i.e., behavioral modeling). Subsequently they received a series of electrical stimuli of the same intensity that were preceded by green or red lights and rated the intensity of the pain elicited by each of the stimuli. The results of this experiment showed that previous observation of the model elicited an analgesic placebo response in observers when the green lights were displayed. Moreover, the pain ratings provided by observers did not correlate with those provided by the model, which suggests that pain reports delivered by the model affected the observers’ subsequent pain experiences rather than just their pain ratings. This study also showed that the magnitude of the observationally induced placebo effect was similar to that produced by classical conditioning and significantly higher than that induced by verbal suggestions. Moreover, it was shown that the empathic concern correlated positively with the magnitude of the observationally induced placebo analgesia.

The findings of Colloca and Benedetti’s (2009) study were confirmed and extended in a series of research. Using the same color lights paradigm and behavioral modeling, Świder and Bąbel (2013) proved that nocebo hyperalgesia may also be acquired through social learning. They also extended the knowledge on the factors affecting observationally induced placebo effects, showing that not only empathic concern but also personal distress in the observer may influence the magnitude of nocebo hyperalgesia. Moreover, they were the first and, so far, the only to have investigated the effect of sex of both the model and observer on the magnitude of nocebo hyperalgesia induced by observational learning. In a detailed examination, they revealed that observation of a male model produced both in women and men a greater nocebo hyperalgesia than observation of a female model.

In their recent study, Świder and Bąbel (2016) replicated the effect discovered by Colloca and Benedetti (2009) by using a modified methodology. They showed that placebo analgesia can be induced by observational learning regardless of the type of stimuli used as placebos. They used colored lights and geometrical shapes as placebos and stated that there were no differences in the magnitudes of placebo effects induced by observational learning when various stimuli served as placebos. In that study, similarly to the previous ones, behavioral modeling was applied.

Vögtle et al. (2013) were the first to investigate the role of symbolic modeling in the context of the nocebo effect. They used a video-clip showing a female model who rated the intensity of pressure pain in two conditions with and without inert ointment and reported more pain when the ointment was applied. Observers who were subsequently exposed to the painful pressure stimulation reacted similarly to the previously observed videotaped model, and when inert ointment was applied they rated the stimuli as more painful than when no ointment was administered. This result showed that observing a live model is not necessary to induce nocebo hyperalgesia as the same effect may be induced by symbolic modeling. Interestingly, there was no significant correlation between empathy and nocebo hyperalgesia induced by the observation of a videotaped model. The authors also highlighted the role of other individual differences in pain modulation. They found a positive correlation between catastrophizing thoughts concerning pain and the magnitude of the nocebo effect.

In their recent study, Vögtle et al. (2016) further investigated possible moderators of nocebo hyperalgesia induced by observation of a videotaped model, such as empathy, pain catastrophizing, somatic, and hypochondriacal concerns. This study partially confirmed previous results showing that empathy was not involved in the nocebo hyperalgesia induced by symbolic modeling; however, no correlation between observationally induced nocebo effect and catastrophizing thoughts as well as other investigated individual characteristics was observed.

Hunter et al. (2014) used the same color lights paradigm as in the study of Colloca and Benedetti (2009) to compare the magnitudes of analgesic placebo effects induced by behavioral modeling (i.e., observation of a live female model) and symbolic modeling (i.e., observation of a videotaped female model). The results of this study revealed that in these two conditions the magnitude of the placebo analgesic effect was similar. Moreover, they clarified findings from previous studies on the role of empathy on observationally induced placebo effects, showing that a positive correlation between analgesic response and emphatic concern occurred only when behavioral modeling was involved. Thus, the authors concluded that empathy is involved in the induction of placebo effects in the observer, but it is not a crucial factor in this process.

The studies cited above proved that observational learning can induce placebo effects. The information derived from observation of pain behaviors displayed by other people may contribute to the formation of expectancies of analgesia or hyperalgesia that may change the chemistry and circuitry of the brain and thereby modulate an individual’s response to pain (Benedetti et al., 2011; Colloca and Miller, 2011; Colloca et al., 2013; Wager and Atlas, 2015). Pain expectancies may be activated in the presence of contextual stimuli (i.e., color light, ointment) which triggered an analgesic or hyperalgesic response in the previously observed model. In all the studies cited above, the effect of pain modulation was triggered by visible and consciously perceptible stimuli. However, recent evidence suggests that observationally induced placebo analgesia may also be activated by subliminally presented stimuli (Egorova et al., 2015).

Egorova et al. (2015) used both classical conditioning and symbolic modeling to induce the placebo effect. During an observational learning session, the participants watched the face of a videotaped model who was being exposed to a series of painful thermal stimuli preceded by the presentation of one of two fractal images. After the presentation of a defined visual stimulus, the model demonstrated a painful grimace or maintained a neutral expression and rated the heat stimuli as painful or non-painful, respectively. The ratings provided by the model were presented on visual analog scales (VASs) displayed on a computer screen. Subsequently the observers were exposed to a series of heat stimuli of moderate intensity preceded by the same two visual stimuli, which were either fully or subliminally visible, i.e., very briefly, and followed by a masking image. The results of the study once again showed that the magnitude of the placebo effect induced observationally and by classical conditioning was similar. Moreover, observationally learned cues, when activated non-consciously, still triggered a robust placebo effect which was resistant to extinction. The results of this study showed that information derived from observation of pain behavior displayed by other people may induce changes in individual pain experiences automatically, without conscious expectancies.

Recent studies have revealed that observation of the model providing pain ratings (i.e., behavioral and symbolic modeling) is not necessary to elicit changes in individual pain experience. It was found that placebo effects may be still induced even when participants had neither direct nor indirect contact with other people experiencing pain, but instead received information about pain ratings provided by a group of people who had supposedly undergone the same painful stimulation previously (i.e., verbal modeling). In the study conducted by Yoshida et al. (2013), the participants watched pain reports provided by eight anonymous people. Pain ratings were marked as bars on a visual analog scale (VAS), where each bar represented the rating of one individual. The presented pain ratings were either above or below the participant’s pain ratings established in the previous, non-manipulated phase of the experiment and were displayed just before the application of pain stimuli. The results of the study showed that this kind of verbal modeling activated thalamocortical regions of the brain which are related to pain. The information provided in this way also affected the individuals’ pain ratings, which were biased toward the mean of the modeled ratings. This means that the participants utilized information from the group and did not conform to the most extreme ratings. However, high variance across modeled pain ratings caused uncertainty about the upcoming pain and largely abolished this bias, substantially increasing the individuals’ pain reports.

The same verbal modeling paradigm was used by Koban and Wager (2016) to investigate the effect of both social information and conditioned visual cues on individual pain reports and skin conductance response (SCR). The participants taking part in this complex experiment experienced heat temperatures preceded by simultaneously presented visual and social cues. Social cues were the pain ratings of ten anonymous people who supposedly experienced the same thermal stimulation. Their ratings were displayed on VASs, depicting either low or high pain ratings. Unlike the visual cues, the social cues were not predictive of the actual stimulus temperature, i.e., both types of ratings were presented equally often for each temperature used in this study. The results of this study revealed that social information was utilized even in the presence of more reliable conditioned cues. Information derived from other’s pain reports had an effect not only on individuals’ pain reports, but also on physiological responses to pain. Moreover, this effect was significantly stronger than that produced by conditioned cues. The study also showed that the effects of both social information and conditioned cues on pain reports and SCRs were fully mediated by expectancies. Moreover, participants with high emphatic concern and optimism were more influenced by social cues, while those with greater reward responsiveness were more influenced by conditioned cues.

In summary, both behavioral and symbolic modeling have been found to induce placebo analgesia and nocebo hyperalgesia, and placebo analgesia induced by either behavioral or symbolic modeling was of similar magnitude to placebo analgesia induced by direct experience, i.e., classical conditioning. It should be also noted that the stimuli learnt by symbolic modeling were found to be strong enough to induce placebo analgesia, even when presented subliminally. Moreover, verbal modeling was also found to induce placebo analgesia, but its effect was fully mediated by expectancies. Studies have also found that trait empathy, especially empathic concern, is a significant factor contributing to both placebo analgesia and nocebo hyperalgesia induced by observational learning when behavioral rather than symbolic modeling is applied. However, empathic concern has also been found to affect the effects of verbal modeling. The sex of the model, catastrophizing thoughts and optimism are also among the factors that have been found to influence the placebo effects induced by observational learning.

It should also be noted that observational learning induced not only changes in pain intensity ratings, but also changes in physiological reactions of the body regulated by the autonomic nervous system, e.g., skin conductance (Koban and Wager, 2016) or heart rate (Colloca and Benedetti, 2009). These results suggest that social cues do not trigger conformity, i.e., matching individual pain judgments to those presented by others, but actually affect individual pain experiences by inducing placebo and nocebo effects.

## Critical Points in Placebo Effects Induced by Observational Learning

Although the studies discussed above significantly contributed to our current knowledge on placebo effects induced by observational learning, they also raised several important issues that need further consideration.

### The Sources of Observational Learning

In studies in which behavioral and symbolic modeling was applied, the models described pain experiences by rating them verbally (Colloca and Benedetti, 2009; Świder and Bąbel, 2013, 2016; Vögtle et al., 2013, 2016) or indicating them on rating scales (Hunter et al., 2014), rather than by physical demonstration of pain behaviors. Moreover, in studies which investigated the effect of behavioral modeling (Colloca and Benedetti, 2009; Świder and Bąbel, 2013, 2016; Hunter et al., 2014), the observers sat next to the model and had limited opportunity to watch his/her facial and body reactions carefully. Similarly, in studies that presented a videotaped model (Vögtle et al., 2013, 2016), the face of the model was only partially visible because the camera was placed at an angle behind the model. Even in the study by Egorova et al. (2015), in which the videotaped model demonstrated painful grimaces and was presented “en face,” the pain ratings provided by the model were explicitly displayed on the screen and it was these pain ratings, rather than facial expressions, that might have been the main source of information concerning the intensity of the pain because they were the most vivid cues. In addition, observers were explicitly instructed to learn the association between presented cues and pain levels, which could have made them focus mainly on presented VASs. Moreover, in a few of the studies the models in fact did not receive any painful stimuli, but pretended to receive pain stimuli (i.e., Świder and Bąbel, 2013, 2016). In those studies, pain reports provided by the model were in fact the only reliable information concerning pain. Thus, the question arises whether verbal modeling rather than behavioral or symbolic modeling was in fact applied in the studies discussed above.

To the best of our knowledge neither placebo analgesia nor nocebo hyperalgesia induced purely by observation of non-verbal behaviors has been systematically investigated, although previous studies on social modeling of pain responses have shown that non-verbal cues related to pain, especially facial cues, might be more trustworthy than verbal pain reports (Craig, 1992; Poole and Craig, 1992; Roy et al., 2015). However, it has been shown that the facial expressions of a model may enhance the magnitude of placebo analgesia induced by classical conditioning (Valentini et al., 2014). The authors used the color lights paradigm and presented the green and red light stimuli before the application of non-painful and painful stimuli, respectively. They complemented this procedure by presenting a videotaped model who displayed painful grimaces, smiles, and neutral facial expressions. Interestingly, the results of that study indicated that watching the facial expressions of both positive and negative emotions significantly enhanced placebo analgesia; however, watching positive emotional expressions resulted in greater placebo analgesia. The authors suggested that the positive emotions expressed by the model might have reinforced observers’ expectations of analgesia triggered by the presentation of stimuli serving as placebos. Although negative emotions expressed by the model which were incongruent to the observers’ expectations might have reduced expectations of relief, they concomitantly distracted the observers’ attention from nociceptive stimulation. In this case placebo analgesia was the result of interaction between attention and expectancies. However, more research is needed to better understand the role of facial expressions of pain as well as the role of other non-verbal cues, i.e., vocal and postural behaviors in modulation of pain experiences.

### Mechanisms of Placebo Effects Induced by Observational Learning

According to the model proposed by Colloca and Miller (2011), observational learning, alongside classical conditioning and verbal suggestions, is one of the means by which placebo effects may be induced. Colloca and Miller (2011) postulate that expectancies are central to the formation of placebo effects induced by social observational learning, as well as verbal suggestions and classical conditioning. This statement is in line with Bandura’s learning theory, which postulates that observational learning results in the acquisition and modification of expectancies (Bandura, 1976). Kirsch (1985) also highlighted that, among other processes (including conditioning and verbal persuasion), modeling is involved in the acquisition and modification of expectancy.

There are many studies showing that expectancies are involved in placebo effects induced by classical conditioning and verbal suggestions (for review see: Kirsch, 1985; Stewart-Williams and Podd, 2004; Peerdeman et al., 2016). There is also some evidence that placebo effects induced by classical conditioning may not necessarily be predicted by conscious and self-reported expectancies (Benedetti et al., 2003; Jensen et al., 2012, 2015; Bąbel et al., 2017a,b). However, little is known about the role of expectancies in placebo effects induced by observational learning. Koban and Wager (2016) were the first and so far the only to have measured expectancies acquired by observational learning. They found that placebo analgesia induced by verbal modeling was fully mediated by expectancies. On the other hand, the results obtained by Egorova et al. (2015) showed that symbolic modeling can even produce the placebo effect non-consciously. This result may suggest that placebo effects induced by observational learning may not necessarily be predicted by conscious expectancies. Therefore, the role of expectancies in the formation of an observationally induced placebo effect requires further investigation.

There is also evidence suggesting that placebo effects may be mediated via activation of emotional processes (Flaten et al., 2011). Data from previous studies have shown that fear, which is related to pain, may be involved in placebo analgesia (de Jong et al., 1996) and nocebo hyperalgesia (Bąbel, 2008; Bąbel et al., 2017b) induced by classical conditioning, as well as placebo analgesia induced by verbal suggestions (Lyby et al., 2012). Previous studies have found that pain-related fear may be acquired through observation (Olsson and Phelps, 2004; Olsson et al., 2007; Helsen et al., 2011). The results of the cited studies show that watching a model experiencing pain and expressing distress in the presence of a defined stimulus can trigger neural and physiological fear responses in the observer when the same stimuli are presented (Olsson and Phelps, 2004; Olsson et al., 2007; Helsen et al., 2011). It can also lead to changes in pain beliefs; however, pain-related beliefs acquired by observing pain in others do not necessarily cause behavioral changes (Helsen et al., 2011, 2015). To the best of our knowledge, the role of fear in the formation of placebo analgesia and nocebo hyperalgesia induced by observational learning has not yet been investigated, although the results of the studies on the role of emotional processes in placebo effects induced by classical conditioning and verbal suggestions imply that fear, alongside expectancy, may be considered another important factor contributing to the placebo effects induced by observational learning.

Neural correlates of direct observational learning on pain experience and placebo analgesia have not been systematically investigated yet. However, previous studies suggest that anterior insula (AI) and anterior cingulate cortex (ACC) are involved in processing of both experienced and observed pain as well as in pain prediction and anticipation. Thus, AI and ACC may be candidates for brain regions responsible for placebo effects induced by observational learning (Koban et al., 2017). There is also data suggesting that mirror processes could be involved in pain modulation induced by observation (Rizzolatti and Fabbri-Destro, 2008; Rizzolatti et al., 2009). Mirror neurons system may help to initiate behaviors and emotions similar to those expressed by the observed model. However, direct evidence for the involvement of mirror neurons in the induction of placebo and nocebo effects through observation is still lacking.

## The Model of Placebo Effects Induced by Observational Learning

Based on Bandura’s social learning theory (Bandura, 1976, 1985) and the results of research investigating placebo effects induced by observational learning, we propose a model which both integrates the existing body of evidence and opens new research perspectives on the mechanisms and factors contributing to placebo effects induced by observational learning.

**FIGURE 1:**
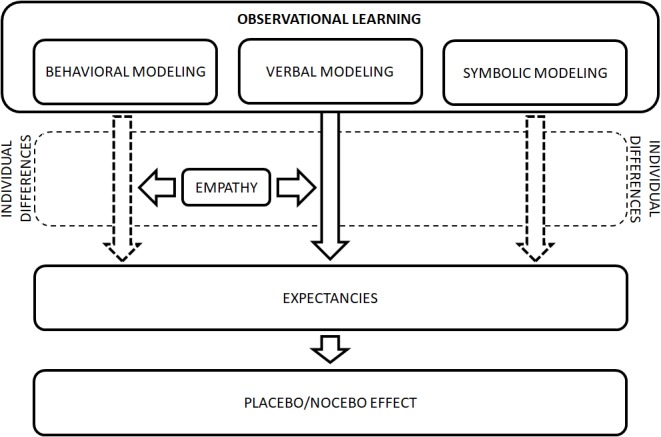
Formation of placebo effects induced by observational learning. Figure depicts the ways of transmission of information concerning pain from the model to the observer: (1) behavioral modeling, (2) verbal modeling, and (3) symbolic modeling. The information conveyed to the observer may contribute to the formation of expectancies of analgesia/hyperalgesia and thereby induce the placebo/nocebo effect. Individual characteristics of the observer, including empathy, are also involved in the formation of observationally induced analgesia and hyperalgesia. The relationships confirmed in previous studies are drawn with a solid line, while those which seem to be possible but are not yet confirmed are depicted with a dashed line.

The model includes three ways of transmission of information concerning pain from the model to the observer: (1) through physical demonstration of pain behavior (behavioral modeling), (2) through verbal description of pain (verbal modeling), and (3) through indirect pictorial representation (symbolic modeling) (Figure 1). The information conveyed to the observer may contribute to the formation of expectancies of analgesia/hyperalgesia and thereby induce the placebo/nocebo effect. Individual characteristics of the observer are also involved in the formation of observationally induced analgesia and hyperalgesia. As most evidence supports the role of empathy in the induction of placebo effects by behavioral and verbal (but not symbolic) modeling, we have included it in the model. However, we assume that other individual characteristics of the observer, including catastrophizing thoughts and optimism, might play a role, but more research is needed to support the existing body of evidence. Although placebo effects induced only by verbal modeling were found to be mediated by expectancies, we hypothesize that expectancies play a role also in placebo effects induced by behavioral and symbolic modeling. Thus, we have included the mediational role of expectancies in the model, although it should be supported by further research. In Figure 1 the relationships confirmed in previous studies are drawn with a solid line, while those which seem to be possible but are not yet confirmed are depicted with a dashed line.

The advantages of the proposed model should be highlighted. This model places the studies on placebo analgesia and nocebo hyperalgesia induced by observational learning in the framework of one of the best-established theories in psychology, i.e., social learning theory (Bandura, 1976, 1985). In effect, existing experimental data have been summarized as well as future findings would be integrated with them within the proposed model. Furthermore, it allows also to uncover the gaps in the current research.

However, there are also some limitations of the proposed model. It integrates data on mechanisms and factors contributing to placebo analgesia and nocebo hyperalgesia induced by observational learning, but may not elucidate mechanisms involved in modulation of other somatic symptoms. Moreover, the model refers to pain induced experimentally and further analysis is needed to extend the scope of the model to clinical pain. Further research is also required to fully test the model, i.e., to reveal the role of expectancies in shaping placebo effects induced by behavioral and symbolic modeling and to determine distinct and combined effect of different types of modeling on the placebo analgesia and nocebo hyperalgesia.

Possible clinical implications of the model should be also acknowledged. The model indicates that individual placebo and nocebo responses may be influenced by information from other patients. This information may be transmitted between patients not only through verbal communication but also through non-verbal behaviors. Moreover, this information may be also provided indirectly, e.g., via internet. This implies that clinicians should draw attention to different aspects of patients’ behavior and control carefully the social variables accompanying the clinical examination and therapy.

## Future Directions

A number of studies have shown that not only pain but also other somatic symptoms may be modulated by observational learning (Lorber et al., 2007; Mazzoni et al., 2010; Broderick et al., 2011; Faasse et al., 2015, 2017). For example, observational learning may be one of the factors behind so-called “mass psychogenic illness,” a form of the nocebo effect which manifests itself in the collective occurrence of physical symptoms in the absence of an identifiable pathogen (Colligan and Murphy, 1982). However, there is also evidence that observation of a model displaying symptoms of relief may cause a similar effect in observers (Faasse et al., 2017). Currently, the main source of information about human behavior is the symbolic environment of the mass media, through which a single model may present a specific reaction to a myriad of people and simultaneously trigger similar reactions in observers (Bandura, 2001). Social network portals and internet forums may be a limitless source of social information which can influence the health behaviors of those who receive them (Moorhead et al., 2013; Laranjo et al., 2015). Therefore, it seems important to intensify studies on the role of symbolic modeling in the formation of placebo and nocebo effects.

As discussed above, placebo effects may be induced by different types of observational learning, i.e., behavioral, symbolic, and verbal modeling. Although behavioral and symbolic modeling seems to induce placebo effects of a similar magnitude (Hunter et al., 2014), it would be valuable to verify whether this is the case when verbal modeling and behavioral or symbolic modeling are compared. Future research should further investigate the cognitive and emotional processes involved in shaping placebo and nocebo effects. They could focus on the role of pain expectancies in the formation of placebo effects. Although the placebo effect induced by verbal modeling is fully mediated by expectancies (Koban and Wager, 2016), it would be important to find out whether placebo effects induced by behavioral and symbolic modeling are also mediated by expectancies. It is also not clear whether the same mechanisms are involved in placebo analgesia and nocebo hyperalgesia induced by observational learning. There is some evidence suggesting that different mechanisms might operate in the case of placebo analgesia and nocebo hyperalgesia, i.e., that classical conditioning might not be as important in nocebo hyperalgesia as in placebo analgesia (Colloca et al., 2008). Thus, whether both placebo analgesia and nocebo hyperalgesia can be induced by all three types of modeling should be determined.

In previous studies the information provided by the models was related to sensory (intensity), but not affective (unpleasantness) characteristics of the pain experience. However, the results of the studies show that these two pain dimensions may be independently processed by different brain regions and may initiate different psychological processes (Rainville et al., 1999; Price, 2000). Thus, the aim of future studies could be to determine whether the information about affective sensations provided by others can modulate individual pain experience and induce placebo effects as effectively as information about sensory sensations.

Research on the personality factors involved in placebo analgesia and nocebo hyperalgesia induced by observational learning should also be continued. It seems that empathy may be one of the most important factors related to observationally induced analgesia or hyperalgesia (Colloca and Benedetti, 2009; Świder and Bąbel, 2013; Hunter et al., 2014; Koban and Wager, 2016); however, the data concerning the role of other individual characteristics that may influence this process are rather inconclusive and this issue needs further investigation. It would also be valuable to investigate traits such as conformity or susceptibility to social influence. The results of the previous studies should also be verified outside of laboratories and should explore the possibility of using social cues in the modulation of clinical pain.

## Author Contributions

EB and PB developed the idea of the paper and accepted the final version of the manuscript. EB drafted the manuscript. PB commented on and extended the first draft.

## Conflict of Interest Statement

The authors declare that the research was conducted in the absence of any commercial or financial relationships that could be construed as a potential conflict of interest.
